# Supplementary material for: A High-Order Cumulant Extension of Quasi-Linkage Equilibrium
Source: ArXiv. 2025 Sep 18:arXiv:2509.10987v2. Preprint. [Version 2] (PMC12458595)
Supplement: Supplement 1 [file NIHPP2509.10987v2-supplement-1.pdf]

Supplementary Information

STOCHASTIC PROCESSES IN POPULATION GENETICS

Following the convention of previous studies [10, 18], we represent genetic sequences as $\boldsymbol{g} \in \{-1, +1\}^L$, where L is the sequence length. This $-/+$ encoding is commonly used in the physics literature. For a genotype $a \in \{1, \ldots, M\}$ with $M = 2^L$, and corresponding sequence $\boldsymbol{g}^a$, the fitness is defined as $F^a = F(\boldsymbol{g}^a)$. We consider the following general form for the fitness function:

$$F(\boldsymbol{g}) = \bar{F} + \sum_i s_i g_i + \sum_{i<j} s_{ij} g_i g_j + \cdots \,, \tag{S1}$$

where the fitness parameters s_i and s_{ij} represent selection coefficients and pairwise epistatic interactions, respectively. Interactions beyond pairwise, such as $s_{ijk}, s_{ijkl}, \ldots$, can also occur, and we refer to these as higher-order epistatic interactions. For simplicity, we denote fitness parameters generically as s_e, where the index e may represent a single-site effect (i), pairwise (i, j), three-way (i, j, k), four-way (i, j, k, l), and so on.

To model the evolution of genetic sequences, we employed the Wright–Fisher (WF) process, a foundational stochastic model in population genetics that captures reproduction dynamics [1]. The WF process describes the evolution of a population with fixed size N. At time t_k (the parent generation), the population is represented by genotype counts $(n_1(t_k), \ldots, n_M(t_k))^\top = (n_a(t_k))_a^M$, which give rise to the next generation at time t_{k+1}, denoted $(n_a(t_{k+1}))_a^M$.

Demographic noise, or genetic drift, arises from stochastic sampling and is on the order of $\mathcal{O}(1/N)$. It can be quantified through fluctuations in genotype frequencies, defined as $\nu_a = n_a/N$. Mathematically, the WF process is a discrete-time multinomial process. Additional evolutionary forces, including selection (with fitness $F(\boldsymbol{g})$), mutation, and recombination, can be incorporated. Mutation and recombination occur at rates μ and r per site per generation, respectively.

Let $\boldsymbol{\nu} = \boldsymbol{n}/N$ denote the genotype frequency vector, $p_a(\boldsymbol{\nu} \mid F, \mu, r)$ denote the probability that genotype a is selected, given the current genotype frequencies $\boldsymbol{\nu}$, fitness function $F(\boldsymbol{g})$, and evolutionary forces such as mutation and recombination (defined below). Then, the WF process can be expressed as:

$$p((\boldsymbol{\nu}(t_k))_{k=0}^K | F, \mu, r, N) = \prod_{k=0}^{K-1} p(\boldsymbol{\nu}(t_{k+1}) | \boldsymbol{\nu}(t_k); F, \mu, r, N) \,, \tag{S2}$$

whre

$$p(\boldsymbol{\nu}(t_{k+1}) | \boldsymbol{\nu}(t_k) \;; F, \mu, r, N) = N! \prod_a \frac{p_a(\boldsymbol{\nu}(t_k) | F, \mu, r)^{N\nu_a(t_{k+1})}}{[N\nu_a(t_{k+1})] \;!} \,. \tag{S3}$$

Let $y_a(\boldsymbol{\nu}; r)$ denote the probability that recombination events produce genotype a, which can be expressed as:

$$y_a(\boldsymbol{\nu}; r) = (1-r)^{L-1} \nu_a + \left(1 - (1-r)^{L-1}\right) \sum_{b,c} R_{a|b,c} \nu_b \nu_c \,. \tag{S4}$$

The selection probability is then given by:

$$p_a(\boldsymbol{\nu} | F, \mu, r) = \frac{y_a(\boldsymbol{\nu}; r) F_a + \mu \sum_{b;\, d_{ab}=1} [y_b(\boldsymbol{\nu}; r) F_b - y_a(\boldsymbol{\nu}; r) F_a]}{\sum_b y_b(\boldsymbol{\nu}; r) F_b} \,. \tag{S5}$$

In this expression, d_{ab} denotes the Hamming distance between genotypes a and b. When the mutation rate μ is low, at most one mutation is expected per individual per generation. As a result, the contribution to the mutation flux in the numerator of (S5) comes only from genotype pairs that differ by a single mutation (i.e., $d_{ab} = 1$).

Details of simulation conditions

To examine a non-trivial scenario, we considered a higher-order fitness function defined as

$$F(\boldsymbol{g}) = \bar{F} + \sum_i s_i g_i + \sum_{i<j} s_{ij} g_i g_j + \sum_{i<j<k} s_{ijk} g_i g_j g_k + \sum_{i<j<k<l} s_{ijkl} g_i g_j g_k g_l \,. \tag{S6}$$

where the fitness coefficients $s_e \in \{-0.03, 0, 0.03\}$ for indices $e = i, (i,j), (i,j,k), (i,j,k,l)$. The number of nonzero coefficients s_e was kept at $\mathcal{O}(L)$ across orders one through four, with L denoting the sequence length. In Fig. 1, to assess the influence of higher-order cumulants on trait dynamics, we also considered random traits defined as

$$G^{\mathrm{Rand}}(\boldsymbol{g}) = \sum_i a_i g_i + \sum_{i<j} a_{ij} g_i g_j \,, \tag{S7}$$

where $a_i \sim \mathcal{N}(0, 1/L)$ and $a_{ij} \sim \mathcal{N}(0, 2/L(L-1))$ for all i, j. These coefficients were independently sampled.

The simulation conditions of **Fig. 1** are as follows. Fitness parameters were drawn from the set $\{-0.03, 0, 0.03\}$, while maintaining the number of nonzero parameters at $O(L)$ for each order, where $L = 100$ is the sequence length. The recombination rate was fixed at $r = 3 \times 10^{-3}$ per site per generation, as the results were robust across different recombination rates. In contrast, the simulation outcomes depend on the mutation rate, which we varied between 10^{-4} and 0.05 per site per generation. The population size was set to $N = 10^3$. As we observed no significant variation in cumulant dynamics after 10^3 generations, we sampled genetic sequences every 200 generations between 10^3 and 2×10^3 generations.

COMPUTATION OF $D_{\mathcal{I},\mathcal{J}}$ MATRIX

We now provide a more explicit expression for $D_{\mathcal{I},\mathcal{J}}$. As we noted in the main text, $\mathcal{I}, \mathcal{J}, \mathcal{K}$ are multi-indices over loci, and cumulants and moments of arbitrary order are defined as $\chi^{\phi}_{\mathcal{I}} = \partial_{\phi_{\mathcal{I}}} \Phi$ and $\mu^{\phi}_{\mathcal{I}} = e^{-\Phi} \partial_{\phi_{\mathcal{I}}} e^{\Phi}$, respectively. The general form of cumulant dynamics is given as

$$\begin{aligned} \partial_{\phi_{\mathcal{I}}} \langle F \rangle_{\boldsymbol{\phi}} |_{\boldsymbol{\phi}=\mathbf{0}} &= \sum_{\mathcal{J}} D_{\mathcal{I},\mathcal{J}} \frac{\partial \langle F \rangle}{\partial \chi_{\mathcal{J}}} \\ D_{\mathcal{I},\mathcal{J}} &= \sum_{\mathcal{K}} \frac{\partial \chi_{\mathcal{J}}}{\partial \mu_{\mathcal{K}}} \left. \frac{\partial \mu^{\phi}_{\mathcal{K}}}{\partial \phi_{\mathcal{I}}} \right|_{\boldsymbol{\phi}=\mathbf{0}} . \end{aligned} \tag{S8}$$

We express moments $\mu_{\mathcal{K}}$ in terms of cumulants since derivatives of cumulants with respect to ϕ are more tractable. Specifically, moments can be written as:

$$\mu_{\mathcal{K}} = \sum_{\pi \in \mathcal{P}(\mathcal{K})} \prod_{B \in \pi} \chi_B \qquad . \tag{S9}$$

where $\mathcal{P}(\mathcal{K})$ denotes all partitions of the index set $\mathcal{K}$. For example, if $\mathcal{K} = \{k_1\}$, then $\mathcal{P}(\mathcal{K}) = \{\{k_1\}\}$. For $\mathcal{K} = \{k_1, k_2\}$, $\mathcal{P}(\mathcal{K}) = \big\{\{k_1, k_2\}, \{\{k_1\}, \{k_2\}\}\big\}$, and so on.

Therefore,

$$\left. \frac{\partial \mu^{\phi}_{\mathcal{K}}}{\partial \phi_{\mathcal{I}}} \right|_{\boldsymbol{\phi}=\mathbf{0}} = \sum_{\pi \in \mathcal{P}(\mathcal{K})} \left[\partial_{\phi_{\mathcal{I}}} \prod_{B \in \pi} \chi^{\phi}_B \right]_{\boldsymbol{\phi}=\mathbf{0}} . \tag{S10}$$

To relate cumulants to moments, we use the Faà di Bruno formula:

$$\chi_{\mathcal{J}} = \sum_{\pi \in \mathcal{P}(\mathcal{J})} (-1)^{|\pi|-1} (|\pi| - 1)! \prod_{B \in \pi} \mu_B \,, \tag{S11}$$

from which, we obtain the derivative of cumulants with respect to moments:

$$\frac{\partial \chi_{\mathcal{J}}}{\partial \mu_{\mathcal{K}}} = \sum_{\pi \in \mathcal{P}(\mathcal{J})} (-1)^{|\pi|-1} (|\pi| - 1)! \frac{\partial}{\partial \mu_{\mathcal{K}}} \prod_{B \in \pi} \mu_B \,. \tag{S12}$$

Thus, combining (S12) and (S10), we obtain the explicit expression of $D_{\mathcal{I},\mathcal{J}}$ in (S8) . Below, we demonstrate this for specific cases under a pairwise fitness function.

$\underline{K = 1 \ case}$

Let us consider a simple $\mathcal{I} = \{i\}\,, \mathcal{J} = \{j\}$ case. The only partition for $\mathcal{K} = \{j\}$ is $\mathcal{P}(\mathcal{K}) = \{\{j\}\}$, and we obtain:

$$\frac{\partial \chi_j}{\partial \mu_{\mathcal{K}}} = \delta_{\{j\},\mathcal{K}}\,,$$

where $\delta_{\mathcal{K},\mathcal{L}}$ returns 1 if $\mathcal{K} = \mathcal{L}$, otherwise returns 0. Thus, the D matrix reduces to:

$$D_{i,j} = \sum_{\mathcal{K}} \delta_{\{j\}\mathcal{K}} \sum_{\pi\in\mathcal{P}(\mathcal{K})} \left[\partial_{\phi_i} \prod_{B\in\pi} \chi_B^{\phi}\right]_{\boldsymbol{\phi}=\mathbf{0}} = \sum_{\mathcal{K}} \delta_{\{j\}\mathcal{K}} \partial_{\phi_i} \chi_j^{\phi}|_{\boldsymbol{\phi}=\mathbf{0}} = \chi_{ij}\,.$$

where only $\mathcal{K} = \{j\}$ contributes. Therefore, this result is consistent with the $K = 1$ case.

$\underline{K = 2 \ case}$

Since the expression for D_{ij} matches the $K = 1$ case, we consider three additional cases: $\mathcal{I} = \{i, j\}$, $\mathcal{J} = \{k\}$; $\mathcal{I} = \{i\}$, $\mathcal{J} = \{k, l\}$; and $\mathcal{I} = \{i, j\}$, $\mathcal{J} = \{k, l\}\,.$

For $\mathcal{I} = \{i, j\}$, $\mathcal{J} = \{k\}$, we have seen that $\frac{\partial \chi_k}{\partial \mu_{\mathcal{K}}} = \delta_{j,k}\,.$ from the example in $K = 1$ case. Therefore, we have

$$D_{ij,k} = \sum_{\mathcal{K}} \delta_{\{k\},\mathcal{K}} \left[\partial_{\phi_i} \partial_{\phi_j} \chi_k^{\phi}\right]_{\boldsymbol{\phi}=\mathbf{0}} = \chi_{ijk}\,.$$

For $\mathcal{I} = \{i\}$, $\mathcal{J} = \{k, l\}$, the cumulant-moment relation yields,

$$\frac{\partial \chi_{kl}}{\partial \mu_{\mathcal{K}}} = -\chi_l \delta_{\{k\},\mathcal{K}} - \chi_k \delta_{\{l\},\mathcal{K}} + \delta_{\{k,l\},\mathcal{K}}\,.$$

Therefore,

$$\begin{aligned} D_{i,kl} &= \sum_{\mathcal{K}} \left(-\chi_l \delta_{\{k\},\mathcal{K}} - \chi_k \delta_{\{l\},\mathcal{K}} + \delta_{\{k,l\},\mathcal{K}}\right) \left[\partial_{\phi_i} \sum_{\pi\in\mathcal{P}(\mathcal{K})} \prod_{B\in\pi} \chi_B^{\phi}\right]_{\boldsymbol{\phi}=\mathbf{0}} \\ &= -\chi_l \chi_{ik} - \chi_k \chi_{il} + \left[\partial_{\phi_i} (\chi_{kl}^{\phi} + \chi_k^{\phi} \chi_l^{\phi})\right]_{\boldsymbol{\phi}=\mathbf{0}} \\ &= -\chi_l \chi_{ik} - \chi_k \chi_{il} + \chi_{ikl} + \chi_l \chi_{ik} + \chi_k \chi_{il} = \chi_{ikl}\,. \end{aligned}$$

For $\mathcal{I} = \{i, j\}$, $\mathcal{J} = \{k, l\}$ case,

$$\begin{aligned} D_{ij,kl} &= \sum_{\mathcal{K}} \left(-\chi_l \delta_{\{k\},\mathcal{K}} - \chi_k \delta_{\{l\},\mathcal{K}} + \delta_{\{k,l\},\mathcal{K}}\right) \left[\partial_{\phi_i} \partial_{\phi_j} \sum_{\pi\in\mathcal{P}(\mathcal{K})} \prod_{B\in\pi} \chi_B^{\phi}\right]_{\boldsymbol{\phi}=\mathbf{0}} \\ &= -\chi_l \chi_{ijk} - \chi_k \chi_{ijl} + \left[\partial_{\phi_i} \partial_{\phi_j} (\chi_{kl}^{\phi} + \chi_k^{\phi} \chi_l^{\phi})\right]_{\boldsymbol{\phi}=\mathbf{0}} \\ &= -\chi_l \chi_{ijk} - \chi_k \chi_{ijl} + \chi_{ijkl} + \chi_{ik} \chi_{jl} + \chi_{il} \chi_{jk} + \chi_l \chi_{ijk} + \chi_k \chi_{ijl} \\ &= \chi_{ijkl} + \chi_{ik} \chi_{jl} + \chi_{il} \chi_{jk}\,. \end{aligned}$$

In summary, we recover the full $D(\boldsymbol{\chi})$ matrix,

$$D(\boldsymbol{\chi}) = \begin{pmatrix} D_{i,k} & D_{i,kl} \\ D_{ij,k} & D_{ij,kl} \end{pmatrix} = \begin{pmatrix} \chi_{ik} & \chi_{ikl} \\ \chi_{ijk} & \chi_{ijkl} + \chi_{ik} \chi_{jl} + \chi_{il} \chi_{jk} \end{pmatrix}\,,$$

consistent with the expression in (9)\,.

DERIVATION OF EXPLICIT EXPRESSIONS FOR FIRST- AND SECOND-ORDER CUMULANTS UNDER PAIRWISE FITNESS FUNCTION

Exact calculation

Here, we derive explicit expressions for the first- and second-order cumulants' equations of motion under the pairwise fitness function and demonstrate that the results derived from the exQLE yield the exact results. Pairwise fitness is defined as:

$$\begin{aligned} F(g) &= \bar{F} + \sum_k s_k g_k + \sum_{k<l} s_{kl} g_k g_l \\ &\bar{F} + \sum_k s_k g_k + \sum_{k<l} s_{kl}(g_k - \chi_k)(g_l - \chi_l) + \sum_{k<l} s_{kl}(g_k - \chi_k)\chi_l + \sum_{k<l} s_{kl}\chi_k(g_l - \chi_l) + \sum_{k<l} s_{kl}\chi_k\chi_l \,. \end{aligned} \tag{S13}$$

Derivatives yield:

$$\begin{aligned} \partial_{\chi_i}\langle F\rangle &= s_i + \sum_{l;l>i} s_{il}\chi_l + \sum_{k;k<i} s_{ki}\chi_k, \\ \partial_{\chi_{ij}}\langle F\rangle &= s_{ij} \,. \end{aligned} \tag{S14}$$

Subtracting,

$$F(g) - \langle F\rangle = \sum_k s_k(g_k - \chi_k) + \sum_{k<l} s_{kl}[(g_k - \chi_k)(g_l - \chi_l) + (g_k - \chi_k)\chi_l + \chi_k(g_l - \chi_l) - \chi_{kl}] \tag{S15}$$

Taking expectations:

$$\begin{aligned} \langle F(g) - \langle F\rangle\rangle &= 0\,, \\ \langle (g_i - \chi_i)(F(g) - \langle F\rangle)\rangle &= \sum_k s_k\chi_{ik} + \sum_{k<l} s_{kl}(\chi_{ikl} + \chi_{ik}\chi_l + \chi_k\chi_{il})\,, \\ \langle (g_i - \chi_i)(g_j - \chi_j)(F(g) - \langle F\rangle)\rangle &= \sum_k s_k\chi_{ijk} + \sum_{k<l} s_{kl}(m_{ijkl} + \chi_{ijk}\chi_l + \chi_k\chi_{ijl} - \chi_{ij}\chi_{kl})\,. \end{aligned} \tag{S16}$$

where the fourth central moment is given by:

$$m_{ijkl} = \chi_{ijkl} + \chi_{ij}\chi_{kl} + \chi_{ik}\chi_{jl} + \chi_{il}\chi_{jk}. \tag{S17}$$

Therefore, the exact equations of motion are:

$$\begin{aligned} \dot{\chi_i} &= \sum_k s_k\chi_{ik} + \sum_{k<l} s_{kl}(\chi_{ikl} + \chi_{ik}\chi_l + \chi_k\chi_{il})\,, \\ \dot{\chi_{ij}} &= \sum_k s_k\chi_{ijk} + \sum_{k<l} s_{kl}(\chi_{ijkl} + \chi_{ik}\chi_{jl} + \chi_{il}\chi_{jk} + \chi_{ijk}\chi_l + \chi_k\chi_{ijl})\,. \end{aligned} \tag{S18}$$

exQLE calculation

As the following calculations are valid for both the Gibbs distribution, and the cumulant distribution of any arbitrary distribution after taking the limit of $\boldsymbol{\phi} \to \mathbf{0}$, we assume that the genotype distribution takes the form of a Gibbs distribution for simplicity. For further simplicity, we drop $\boldsymbol{\phi}$ from $\chi_i^{\boldsymbol{\phi}}$, $\chi_{ij}^{\boldsymbol{\phi}}$, and also omit the operation of taking the limit as $\boldsymbol{\phi} \to \mathbf{0}$. The equation of motion for the first-order cumulant (7) is straightforward to obtain, using the relationships $\partial_{\phi_i}\chi_k = \chi_{ik}$ and $\partial_{\phi_i}\chi_{kl} = \chi_{ikl}$, which arise from the properties of the cumulant generating function or the normalization of the Gibbs distribution.

Here, we focus on the equation of motion for the second-order cumulant. The direct calculation of the exQLE for

the second-order cumulants yields (8), which is given as:

$$\begin{aligned}
\partial_{\phi_i}\partial_{\phi_j}\langle F\rangle &= \partial_{\phi_j}\left(\sum_k(\partial_{\phi_i}\chi_k)\partial_{\chi_k}\langle F\rangle + \sum_{k<l}(\partial_{\phi_i}\chi_{kl})\partial_{\chi_{kl}}\langle F\rangle\right) \\
&= \sum_k(\partial_{\phi_j}\partial_{\phi_i}\chi_k)\partial_{\chi_k}\langle F\rangle + \sum_{k<l}(\partial_{\phi_j}\partial_{\phi_i}\chi_{kl})\partial_{\chi_{kl}}\langle F\rangle + \sum_k(\partial_{\phi_i}\chi_k)\partial_{\phi_j}\partial_{\chi_k}\langle F\rangle + \sum_{k<l}(\partial_{\phi_i}\chi_{kl})\cancelto{0}{\partial_{\phi_j}\partial_{\chi_{kl}}\langle F\rangle} \\
&= \sum_k \chi_{ijk}\ \partial_{\chi_k}\langle F\rangle + \sum_{k<l}\ \chi_{ijkl}\partial_{\chi_{kl}}\langle F\rangle + \sum_k \chi_{ik}\left(\sum_l \chi_{jl}\partial_{\chi_l}\partial_{\chi_k}\langle F\rangle + \sum_{l<m}\chi_{jlm}\cancelto{0}{\partial_{\chi_{lm}}\partial_{\chi_k}\langle F\rangle}\right) \\
&= \sum_k \chi_{ijk}\ \partial_{\chi_k}\langle F\rangle + \sum_{k<l}\ (\chi_{ijkl} + \chi_{ik}\chi_{jl} + \chi_{il}\chi_{jk})\partial_{\chi_{kl}}\langle F\rangle \,.
\end{aligned} \tag{S19}$$

Here, we used the fact that the derivative of the average fitness beyond the second-order cumulant vanishes. Additionally we used: $\partial_{\chi_k}\partial_{\chi_l}\langle F\rangle = \partial_{\chi_{kl}}\langle F\rangle$ and

$$\sum_{k,l}\chi_{ik}\chi_{jl}\partial_{\chi_{kl}}\langle F\rangle = \sum_{k<l}(\chi_{ik}\chi_{jl} + \chi_{il}\chi_{jk})\partial_{\chi_{kl}}\langle F\rangle \,. \tag{S20}$$

The equations of motion for exQLE, corresponding to (7) and (8), lead to:

$$\begin{aligned}
\dot{\chi_i} &= \sum_k \chi_{ik}\partial_{\chi_k}\langle F\rangle + \sum_{k<l}\chi_{ikl}\partial_{\chi_{kl}}\langle F\rangle \\
&= \sum_k \chi_{ik}\left(s_k + \sum_{l;l>k} s_{kl}\chi_l + \sum_{l;l<k} s_{lk}\chi_l\right) + \sum_{k<l} s_{kl}\chi_{ikl} \\
&= \sum_k \chi_{ik}s_k + \sum_{k<l} s_{kl}(\chi_{ik}\chi_l + \chi_{il}\chi_k + \chi_{ikl}) \,,
\end{aligned} \tag{S21}$$

and

$$\begin{aligned}
\dot{\chi_{ij}} &= \sum_k \chi_{ijk}\partial_{\chi_k}\langle F\rangle + \sum_{k<l}(\chi_{ijkl} + \chi_{ik}\chi_{jl} + \chi_{il}\chi_{jk})\partial_{\chi_{kl}}\langle F\rangle \\
&= \sum_k \chi_{ijk}\left(s_k + \sum_{l;l>k} s_{kl}\chi_l + \sum_{l;l<k} s_{lk}\chi_l\right) + \sum_{k<l} s_{kl}(\chi_{ijkl} + \chi_{ik}\chi_{jl} + \chi_{il}\chi_{jk}) \\
&= \sum_k s_k\chi_{ijk} + \sum_{k<l} s_{kl}(\chi_{ijk}\chi_l + \chi_{ijl}\chi_k + \chi_{ijkl} + \chi_{ik}\chi_{jl} + \chi_{il}\chi_{jk}) \,.
\end{aligned} \tag{S22}$$

Here, where we used:

$$\sum_k \chi_{ik}\sum_{l;l<k} s_{lk}\chi_l = \sum_{k<l} s_{kl}\chi_{il}\chi_k \,, \tag{S23}$$

by simply renaming indices. The two sets of equations are identical.

Therefore, we directly confirmed that the equations of motion for the first and second cumulants, derived from the exact Price's equation and the exQLE equation, yield identical results in the case of pairwise fitness.

PROOF OF POSITIVE SEMIDEFINITENESS OF D MATRIX FOR $K \in \{1, 2\}$

For the $K = 1$ case, $D(\boldsymbol{\chi})$ is trivially positive semidefinite. For $K = 2$ case, by defining $\Delta_i := g_i - \chi_i$, it can be expressed as

$$\begin{aligned} D(\boldsymbol{\chi}) &= \begin{pmatrix} \chi_{ik} & \chi_{ikl} \\ \chi_{ijk} & \chi_{ijkl} + \chi_{ik}\chi_{jl} + \chi_{il}\chi_{jk} \end{pmatrix} \\ &= \left\langle \begin{pmatrix} \Delta_i\Delta_j & \Delta_i\Delta_k\Delta_l \\ \Delta_i\Delta_j\Delta_k & (\Delta_i\Delta_j - \chi_{ij})(\Delta_k\Delta_l - \chi_{kl}) \end{pmatrix} \right\rangle \\ &= \left\langle \begin{pmatrix} \Delta_i \\ \Delta_i\Delta_j - \chi_{ij} \end{pmatrix} \begin{pmatrix} \Delta_k \\ \Delta_k\Delta_l - \chi_{kl} \end{pmatrix}^\top \right\rangle \succeq 0\,. \end{aligned} \tag{S24}$$

From the second line to the third line, we used the fact $\langle \Delta_i\Delta_j\Delta_k \rangle = \langle (\Delta_i\Delta_j - \chi_{ij})\Delta_k \rangle$. Therefore, $D(\boldsymbol{\chi})$ is positive semidefinite.

DERIVATION OF THE CUMULANT DYNAMICS FROM GENOTYPE DYNAMICS

We denote the genotype distribution $P(\boldsymbol{g})$ for $\boldsymbol{g} \in \{-1, 1\}^L$ as $P(\boldsymbol{g}^a) \mapsto z_a$, where each unique genotype is indexed by $a \in \{1, \ldots, 2^L\}$. We also denote genotype-level selection coefficient as h_a, such that the fitness function satisfies $F(\boldsymbol{g}^a) = \bar{F} + h_a$.

The average fitness is then given by

$$\langle F \rangle = \bar{F} + \sum_a h_a z_a = \bar{F} + \sum_{\mathcal{J}} s_{\mathcal{J}} \mu_{\mathcal{J}}\,. \tag{S25}$$

Here, $\mu_{\mathcal{J}}$ denote a moment indexed by the set $\mathcal{J}$ (which may include multiple indices), and given by

$$\mu_{\mathcal{J}} = \sum_a z_a \prod_{j \in \mathcal{J}} g_j^a\,. \tag{S26}$$

Let define the matrix,

$$\mathcal{G}_{a,\mathcal{J}} = \prod_{j \in \mathcal{J}} g_j^a \qquad , \tag{S27}$$

so that the moments and genotype distributions are related via $\boldsymbol{\mu} = \mathcal{G}^\top \boldsymbol{z}$.

Given Fisher's fundamental theorem, the genotype distribution evolves as

$$\dot{z}_a = \sum_{ab} C_{ab}(\boldsymbol{z}) h_b \tag{S28}$$

where

$$C_{ab}(\boldsymbol{z}) = z_a \delta_{a,b} - z_a z_b\,, \tag{S29}$$

is the covariance matrix of genotype frequencies. In the vector form, it can be expressed as $\dot{\boldsymbol{z}} = C(\boldsymbol{z})\boldsymbol{h} = C(\boldsymbol{z})\boldsymbol{\nabla}_{\boldsymbol{z}}\langle F \rangle$.

Since $\langle F \rangle$ depends on $\boldsymbol{z}$ via the moments $\boldsymbol{\mu}$, we apply the chain rule:

$$\frac{\partial \langle F \rangle}{\partial z_a} = \sum_{\mathcal{J}} \frac{\partial \mu_{\mathcal{J}}}{\partial z_a} \frac{\partial \langle F \rangle}{\partial \mu_{\mathcal{J}}} = \sum_{\mathcal{J}} \mathcal{G}_{a,\mathcal{J}} \frac{\partial \langle F \rangle}{\partial \mu_{\mathcal{J}}}\,, \tag{S30}$$

which can be expressed in vector form $\boldsymbol{\nabla}_{\boldsymbol{z}}\langle F \rangle = \mathcal{G}\boldsymbol{\nabla}_{\boldsymbol{\mu}}\langle F \rangle$.

Substituting into the dynamics:

$$\dot{\boldsymbol{z}} = C(\boldsymbol{z})\mathcal{G}\boldsymbol{\nabla}_{\boldsymbol{\mu}}\langle F \rangle\,. \tag{S31}$$

Using the relation $\boldsymbol{\mu} = \mathcal{G}^\top \boldsymbol{z}$, the moment dynamics become

$$\dot{\boldsymbol{\mu}} = \mathcal{G}^\top C(\boldsymbol{z})\mathcal{G}\boldsymbol{\nabla}_{\boldsymbol{\mu}}\langle F \rangle\,, \tag{S32}$$

which can be further transformed by using the chain rule:

$$\dot{\boldsymbol{\mu}} = \mathcal{G}^\top C(\boldsymbol{z})\mathcal{G}\left(\boldsymbol{\nabla}_{\boldsymbol{\mu}}\boldsymbol{\chi}^\top\right)\boldsymbol{\nabla}_{\boldsymbol{\chi}}\langle F\rangle\,. \tag{S33}$$

Transforming from moments to cumulants using the Jacobian matrix: $\mathrm{d}\boldsymbol{\mu} = \left(\boldsymbol{\nabla}_{\boldsymbol{\chi}}\boldsymbol{\mu}^\top\right)^\top \mathrm{d}\boldsymbol{\chi}$. Substituting this into the dynamics gives: $\dot{\boldsymbol{\mu}} = \left(\boldsymbol{\nabla}_{\boldsymbol{\chi}}\boldsymbol{\mu}^\top\right)^\top \dot{\boldsymbol{\chi}}$.

By using the relation,

$$\left(\left(\boldsymbol{\nabla}_{\boldsymbol{\chi}}\boldsymbol{\mu}^\top\right)^\top\right)^{-1} = \left(\left(\boldsymbol{\nabla}_{\boldsymbol{\chi}}\boldsymbol{\mu}^\top\right)^{-1}\right)^\top = \left(\boldsymbol{\nabla}_{\boldsymbol{\mu}}\boldsymbol{\chi}^\top\right)^\top,$$

we obtain the cumulant dynamics:

$$\dot{\boldsymbol{\chi}} = D(\boldsymbol{\chi})\boldsymbol{\nabla}_{\boldsymbol{\chi}}\langle F\rangle\,, \tag{S34}$$

where

$$D(\boldsymbol{\chi}) = \left(\boldsymbol{\nabla}_{\boldsymbol{\mu}}\boldsymbol{\chi}^\top\right)^\top \mathcal{G}^\top C(\boldsymbol{z})\mathcal{G}\left(\boldsymbol{\nabla}_{\boldsymbol{\mu}}\boldsymbol{\chi}^\top\right)\,. \tag{S35}$$

This is the matrix $D(\boldsymbol{\chi})$ appearing in (6) of the main text. Its origin lies in $C(\boldsymbol{z})$, which arises from competition between genotypes and also serves as the covariance matrix in the diffusion process. Importantly, since $C(\boldsymbol{z})$ is symmetric and positive semidefinite, the matrix $D(\boldsymbol{\chi})$ inherits these properties, it is symmetric and positive semidefinite as well.

DERIVATION OF THE EQUATION TO INFER FITNESS VALUES

We now derive the equation used to infer fitness parameters from cumulant dynamics.

The cumulant dynamics described in (9) have been deterministic, assuming an infinitely large population. For a finite population of size N, however, stochastic effects must be considered. In this case, the dynamics become a Langevin equation:

$$\dot{\boldsymbol{\chi}} = D(\boldsymbol{\chi})\boldsymbol{\nabla}_{\boldsymbol{\chi}}\langle F\rangle + \sqrt{D(\boldsymbol{\chi}(t))/N}\,\boldsymbol{\eta}(t)\,. \tag{S36}$$

where $\boldsymbol{\eta}(t)$ is a noise vector satisfying $\langle\boldsymbol{\eta}\rangle = \mathbf{0}$ and $\langle\eta_{\mathcal{J}}(t)\eta_{\mathcal{K}}(t')\rangle = \delta_{\mathcal{J},\mathcal{K}}\delta(t-t')$.

This Langevin equation is equivalent to the following Fokker–Planck equation [78]:

$$\partial_t P(\boldsymbol{\chi},t) = -\boldsymbol{\nabla}_{\boldsymbol{\chi}}^\top D(\boldsymbol{\chi})\boldsymbol{\nabla}_{\boldsymbol{\chi}}\langle F\rangle P(\boldsymbol{\chi},t) + \frac{N}{2}\operatorname{Tr}\left(\boldsymbol{\nabla}_{\boldsymbol{\chi}}\boldsymbol{\nabla}_{\boldsymbol{\chi}}^\top D(\boldsymbol{\chi})\right)P(\boldsymbol{\chi},t)\,. \tag{S37}$$

The Fokker–Planck equation can be rewritten as a probability density over entire cumulant trajectories. For numerical implementation, we discretize time at points t_k for $k \in \{0, 1, \ldots, K+1\}$, and define $\Delta t_k := t_{k+1} - t_k$ and $\boldsymbol{\Delta\chi}(t_k) := \boldsymbol{\chi}(t_{k+1}) - \boldsymbol{\chi}(t_k)$.

The probability of a cumulant trajectory is then expressed as

$$P((\boldsymbol{\chi}(t_k))_{k=0}^{K+1}) \propto e^{-N\mathcal{S}((\boldsymbol{\chi}(t_k))_{k=0}^{K+1})}\,, \tag{S38}$$

where $\mathcal{S}$ is given by

$$\begin{aligned}\mathcal{S}((\boldsymbol{\chi}(t_k))_{k=0}^{K+1}) = \sum_{k=0}^{K}\frac{1}{2\Delta t_k}&\Big[\boldsymbol{\Delta\chi}(t_k) - \Delta t_k D(\boldsymbol{\chi}(t_k))\boldsymbol{\nabla}_{\boldsymbol{\chi}}\langle F\rangle\Big]^\top \\ &\times D^{-1}(\boldsymbol{\chi}(t_k))\Big[\boldsymbol{\Delta\chi}(t_k) - \Delta t_k D(\boldsymbol{\chi}(t_k))\boldsymbol{\nabla}_{\boldsymbol{\chi}}\langle F\rangle\Big]\,.\end{aligned} \tag{S39}$$

Since $\mathcal{S}$ is quadratic in $\boldsymbol{\nabla}_{\boldsymbol{\chi}}\langle F\rangle$, the maximum likelihood estimate of the fitness parameters can be obtained analytically by solving the following linear equation:

$$\sum_{k=0}^{K}\Delta\boldsymbol{\chi}(t_k) = \sum_{k=0}^{K}\Delta t_k D(\boldsymbol{\chi}(t_k))\boldsymbol{\nabla}_{\boldsymbol{\chi}}\langle F\rangle\,. \tag{S40}$$

By solving the above maximum likelihood equation, which is linear in $\boldsymbol{s}$, we can obtain $\boldsymbol{s}$.

EFFICIENT COMPUTATION OF THE FORWARD EXQLE SIMULATION UNDER HIGHER-ORDER FITNESS

To obtain cumulant dynamics using either the exQLE framework or the exQLE with a Gaussian closure scheme, we must evaluate the products of the diffusion matrix, which involve third and fourth-order cumulants and fitness parameters. However, a direct computation of the diffusion matrix and its product with fitness parameters is computationally expensive. Estimating these values across multiple time points, on the order of 100 different time points, requires significant computational time. Additionally, incorporating a fitness function that depends on four-way interactions further increases computational complexity. To improve computational efficiency, we outline an optimized approach for computing cumulant dynamics.

Denote the gradient of the average fitness, which serves as the effective fitness parameter vector consisting of selection and epistatic coefficients, as $\begin{pmatrix}\hat{s}_k\\ \hat{s}_{kl}\end{pmatrix} := \begin{pmatrix}\partial_{\chi_k}\\ \partial_{\chi_{kl}}\end{pmatrix}\langle F\rangle$. For the fitness function with four-way interactions, let $\mu_i, \mu_{ij}, \ldots$ denote mutation frequencies, and let $\langle F\rangle = \bar{F} + \sum_i s_i\mu_i + \sum_{i<j} s_{ij}\mu_{ij} + \sum_{i<j<k} s_{ijk}\mu_{ijk} + \sum_{i<j<k<l} s_{ijkl}\mu_{ijkl}$ represent the effective fitness parameters as

$$\begin{pmatrix}\hat{s}_k\\ \hat{s}_{kl}\end{pmatrix} = \begin{pmatrix} s_k + \sum_{i(<k)} s_{ik}\mu_i + \sum_{i<j;\ <k} s_{ijk}\mu_{ij} + \sum_{i<j<l\ ;<k} s_{ijlk}\mu_{ijl} \\ s_{kl} + \sum_{i;\ <k<l} s_{ikl}\mu_i + \sum_{i<j;\ <k<l} s_{ijkl}\mu_{ij}\end{pmatrix}. \tag{S41}$$

These products between fitness parameters and moments can be computed as the sum of matrix-vector products, $s_{ijk}\mu_{ij} = \sum_{i<j(<k)}\langle s_{ijk}g_ig_j\rangle$ and $s_{ijlk}\mu_{ijl} = \sum_{i<j<l(<k)}\langle s_{ijlk}g_ig_jg_l\rangle$.

Let $\Delta_i := g_i - \chi_i$, then the cumulants can be obtained by $\chi_i = \langle\Delta_i\rangle, \chi_{ij} = \langle\Delta_i\Delta_j\rangle, \chi_{ijk} = \langle\Delta_i\Delta_j\Delta_k\rangle, \chi_{ijkl} = \langle\Delta_i\Delta_j\Delta_k\Delta_l\rangle - (\chi_{ik}\chi_{jl} + \chi_{il}\chi_{jk} + \chi_{ij}\chi_{kl})$. Additionally, define $\chi^{(2)}$ as the second-order cumulant in matrix form and $\hat{S}$ as the effective epistasis matrix, where $\hat{s}_{kl}$ occupies the k-th row and l-th column. Thus, the cumulant dynamics can be expressed in the following computationally more efficient form:

$$\begin{pmatrix}\dot{\chi}_i\\ \dot{\chi}_{ij}\end{pmatrix} = \begin{pmatrix}\chi_{ik} & \chi_{ikl}\\ \chi_{ijk} & \chi_{ijkl} + \chi_{ik}\chi_{jl} + \chi_{il}\chi_{jk}\end{pmatrix}\begin{pmatrix}\hat{s}_k\\ \hat{s}_{kl}\end{pmatrix} = \begin{pmatrix}\left\langle\boldsymbol{\delta}(\boldsymbol{\Delta}^\top\hat{\boldsymbol{s}})\right\rangle + \left\langle\boldsymbol{\Delta}(\boldsymbol{\Delta}^\top\hat{S}\boldsymbol{\Delta})\right\rangle/2 \\ \left\langle\boldsymbol{\Delta}\boldsymbol{\Delta}^\top(\boldsymbol{\Delta}^\top\hat{\boldsymbol{s}})\right\rangle + \left\langle\boldsymbol{\Delta}\boldsymbol{\Delta}^\top\boldsymbol{\Delta}^\top\hat{S}\boldsymbol{\Delta}\right\rangle - \chi^{(2)}\mathrm{Sum}\left(\chi^{(2)}\odot\hat{S}\right)\end{pmatrix}. \tag{S42}$$

The last expression in (S42) is efficient because this computation never explicitly requires obtaining matrices or matrix products with more than $\mathcal{O}(L^2)$ elements.

For the Gaussian closure scheme, the cumulant dynamics can be obtained in the same manner. The dynamics under the Gaussian closure scheme are given by:

$$\begin{aligned}\begin{pmatrix}\dot{\chi}_i\\ \dot{\chi}_{ij}\end{pmatrix} &= \begin{pmatrix}\chi_{ik} & \chi_{ikl}(\delta_{ik}+\delta_{jk})\\ \chi_{ijk}(\delta_{ik}+\delta_{jk}) & \chi_{ijkl}\delta_{ik}\delta_{jl} + \chi_{ik}\chi_{jl} + \chi_{il}\chi_{jk}\end{pmatrix}\begin{pmatrix}\hat{s}_k\\ \hat{s}_{kl}\end{pmatrix}\\ &= \begin{pmatrix}\chi_{ik} & \chi_{ikl}(\delta_{ik}+\delta_{jk})\\ \chi_{ijk}(\delta_{ik}+\delta_{jk}) & [\langle\Delta_i\Delta_j\Delta_i\Delta_j\rangle - \chi_{ii}\chi_{jj} - 2\chi_{ij}^2]\delta_{ik}\delta_{jl} + \chi_{ik}\chi_{jl} + \chi_{il}\chi_{jk}\end{pmatrix}\begin{pmatrix}\hat{s}_k\\ \hat{s}_{kl}\end{pmatrix}\\ &= \begin{pmatrix}\left\langle\boldsymbol{\Delta}(\boldsymbol{\Delta}^\top\hat{\boldsymbol{s}})\right\rangle + \left\langle\boldsymbol{\Delta}\odot\left(\sum_k\left\{\left(\boldsymbol{\Delta}\boldsymbol{\Delta}^\top\odot\hat{S}\right)_{ik} + \left(\boldsymbol{\Delta}\boldsymbol{\Delta}^\top\odot\hat{S}\right)_{ki}\right\}\right)_{i=1}^{L}\right\rangle \\ \left\langle\boldsymbol{\Delta}\boldsymbol{\Delta}^\top\odot\left(\mathbf{1}(\boldsymbol{\Delta}\odot\hat{\boldsymbol{s}})^\top + (\boldsymbol{\Delta}\odot\hat{\boldsymbol{s}})\mathbf{1}^\top\right)\right\rangle + \mathrm{diag}\left(\left(\left(\langle\Delta_i^2\Delta_j^2\rangle - \chi_{ii}\chi_{jj} - 2\chi_{ij}^2\right)s_{ij}\right)_{i<j}\right) + \chi^{(2)}\hat{S}\chi^{(2)}\end{pmatrix},\end{aligned} \tag{S43}$$

where $\mathbf{1}$ is a vector of length L consisting entirely of ones, $\odot$ denotes the elementwise product, and the notation $(a_i)_i^L = (a_1, \ldots, a_L)^\top$ is used.

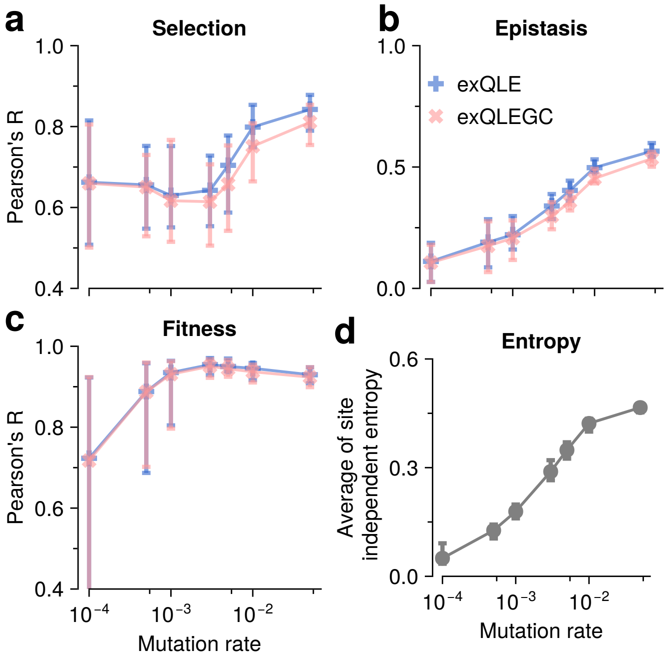

FIG. S1. **Accuracy of inferred fitness parameters under higher-order fitness function.** This corresponds to **Fig. 2** in the main text, but here the underlying selective pressure is determined by a higher-order fitness function rather than a pairwise one, that is, $K^* = 4$, where K^* denotes the highest order of cumulants in the averaged fitness function (as defined in the main text). The functionality and model parameters are the same as those used in the simulation for **Fig. 1**. The inference approach remains pairwise ($K = 2$), aiming to infer additive (selection) and pairwise (epistatic) fitness parameters. Despite the increased complexity of the true fitness landscape, the inferred parameters show high accuracy, as measured by Pearson's R values, for selection, epistasis, and overall fitness. The dependency of accuracy on mutation rate remains qualitatively similar to that observed under the pairwise fitness setting.

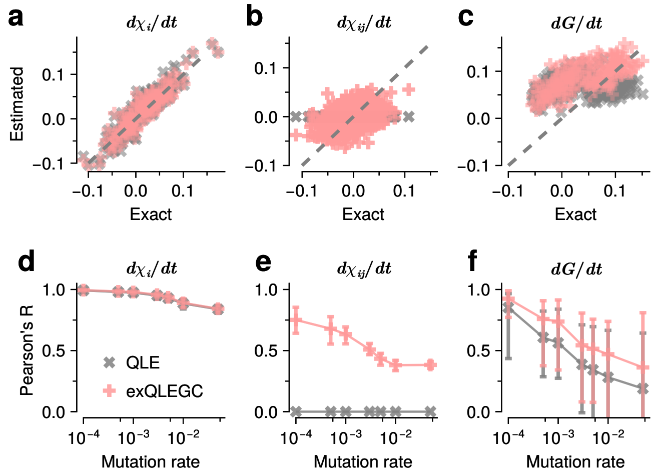

FIG. S2. **Dynamics of cumulants and traits using exQLE and Gaussian closure scheme.** This corresponds to **Fig. 1** in the main text but utilizes the exQLE framework with a Gaussian closure (GC) scheme, which suppresses all cumulants beyond second order. The GC scheme is efficient for inferring fitness parameters and is not limited to inference problems; it is also applicable to forward processes. Although the accuracy of the dynamics for cumulants (**b**) is not as high as that in the exQLE case without GC, the estimated values show a reasonable correlation with those from the exact calculations. Pearson's R values for additive selection (**a**), pairwise epistasis (**b**), and random traits (**c**) for exQLE with GC are $0.93, 0.43$, and 0.65. For comparison, the exQLE values without GC are $0.97, 0.89$, and 0.95, respectively.
